# Combined analysis of DNA methylome and transcriptome reveal novel candidate genes with susceptibility to bovine *Staphylococcus aureus* subclinical mastitis

**DOI:** 10.1038/srep29390

**Published:** 2016-07-14

**Authors:** Minyan Song, Yanghua He, Huangkai Zhou, Yi Zhang, Xizhi Li, Ying Yu

**Affiliations:** 1 Key Laboratory of Animal Genetics, Breeding and Reproduction, Ministry of Agriculture & National Engineering Laboratory for Animal Breeding, College of Animal Science and Technology, China Agricultural University, 100193, Beijing, P.R. China; 2 Department of Animal & Avian Sciences, University of Maryland, College Park, Maryland, 20742, USA; 3 Guangzhou Genedenovo Biotechnology Co., Ltd., Guangzhou, China; 4 Beijing Sanyuan Breeding Technology Co. Ltd., Capital Agribusiness Group, Beijing, China

## Abstract

Subclinical mastitis is a widely spread disease of lactating cows. Its major pathogen is *Staphylococcus aureus* (*S. aureus*). In this study, we performed genome-wide integrative analysis of DNA methylation and transcriptional expression to identify candidate genes and pathways relevant to bovine *S. aureus* subclinical mastitis. The genome-scale DNA methylation profiles of peripheral blood lymphocytes in cows with *S. aureus* subclinical mastitis (SA group) and healthy controls (CK) were generated by methylated DNA immunoprecipitation combined with microarrays. We identified 1078 differentially methylated genes in SA cows compared with the controls. By integrating DNA methylation and transcriptome data, 58 differentially methylated genes were shared with differently expressed genes, in which 20.7% distinctly hypermethylated genes showed down-regulated expression in SA versus CK, whereas 14.3% dramatically hypomethylated genes showed up-regulated expression. Integrated pathway analysis suggested that these genes were related to inflammation, ErbB signalling pathway and mismatch repair. Further functional analysis revealed that three genes, *NRG1*, *MST1* and *NAT9*, were strongly correlated with the progression of *S. aureus* subclinical mastitis and could be used as powerful biomarkers for the improvement of bovine mastitis resistance. Our studies lay the groundwork for epigenetic modification and mechanistic studies on susceptibility of bovine mastitis.

Bovine mastitis, the inflammation of the mammary gland, is one of the most common diseases of dairy cattle. Mastitis is responsible for the rate of elimination, low milk yield and poor milk quality; therefore, it induces significant economic losses in the dairy cattle industry^1^. The disease has distinct importance in public health because of the indiscriminate use of antibiotics and the risk of antibiotic residues through milk consumption^2^. Bovine mastitis is normally divided into subclinical and clinical mastitis; the incidence of subclinical mastitis is much higher (25–65% worldwide) than that of clinical mastitis (normally less than 5%)^3^. *Staphylococcus aureus*, a Gram-positive pathogenic bacterium, is a major subclinical mastitis-causing pathogen in dairy cattle. Bovine mastitis naturally infected by *S. aureus* is usually asymptomatic, persistent, resistant to antibiotic treatment and easily reoccurs^4^,^5^,^6^. More seriously, *S. aureus* often evades host immune response systems to invade and survive in diverse cell types, including mammary epithelial cells, neutrophils, macrophages and peripheral blood lymphocytes^7^,^8^.

To date, a large number of studies have focused on the investigation of virulence factors involved in the pathogenesis of *S. aureus* infection and the host transcriptional responses to *S. aureus*^9^,^10^,^11^. The results of the conducted studies revealed that gene expression greatly changes after *S. aureus* infection^12^,^13^. However, the epigenetic profile and regulatory pathways of bovine subclinical mastitis infected by *S. aureus* remain undefined.

DNA methylation is one of the central epigenetic modifications in most eukaryote genomes and plays an important role in genome-wide pre-transcriptional regulation. Furthermore, the epigenetic mark links the interactions among genetic, epigenetic and environmental factors, and it is associated with many biological processes, such as transcriptional silencing, X-chromosome inactivation, genomic imprinting, inflammation and carcinogenesis^14^. DNA methylation on the promoter or the first exon of a gene generally leads to transcriptional silencing^15^,^16^. The genome-wide DNA methylation map is important to understand changes in DNA methylation during disease progression. The genome-wide DNA methylation map of many species, such as human (12 tissues and melanoma cell strains)^17^,^18^, cattle (placentas)^19^, pig (adipose and muscle)^20^, sheep (muscle)^21^ and rat (lung)^22^, has been reported. In bovine, the potential prognostic value of promoter hypermethylation of the *αs1-casein* gene has been demonstrated in mammary gland tissues of dairy cows for acute mastitis induced by *Escherichia coli*^23^. The DNA methylation level of the *CD4* gene promoter is strongly influenced by the mastitis status in Holstein samples, so it can be used as a powerful epigenetic marker for clinical mastitis in dairy cows^24^. However, the genome-wide DNA methylation regulation of bovine subclinical mastitis naturally induced by *S. aureus* remains unknown.

The present study aimed to document the landscape of DNA methylome distribution in the bovine peripheral lymphocyte genome of Holsteins with subclinical mastitis and naturally infected by *S. aureus*, as well as to analyse potential DNA methylation targets related to host response and resistance to *S. aureus* subclinical mastitis. Finally, we found three novel DNA methylation target genes (*MST1, NRG1* and *NAT9*) that were strongly correlated with susceptibility of *S. aureus* subclinical mastitis in Holstein cows.

## Results

### Identification of subclinical mastitis in Holsteins naturally infected by *S. aureu*s

**Figure 1:**
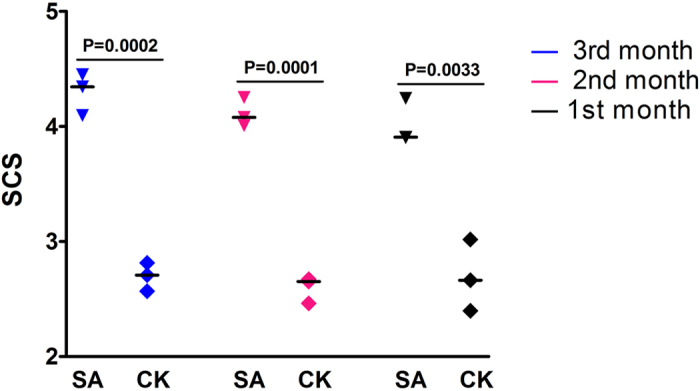
Individual milk SCS of three consecutive months before sampling of the SA and CK cows. SA: Subclinical mastitis cows that were naturally infected with *S. aureus*, CK: healthy cows that were not infected with *S. aureus*. Blue indicates the SCS checked at three months before sampling; red indicates the SCS at two months before sampling; black indicates the SCS checked at the last month before sampling. *P* < 0.01 means a highly significant difference between the SA and CK groups.

**Figure 2:**
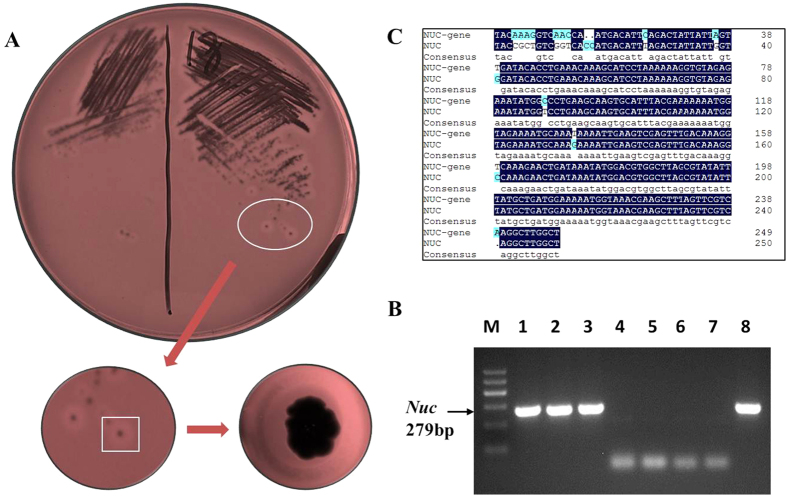
Culture and identification of *S. aureus* separated from the milk of samples. (**A**) Positive sample infected by *S. aureus* in Baird–Parker agar. The *S. aureus* single colony was amplified. (**B**) Specific PCR and electrophoresis map of the *Nuc* gene. Lanes 1, 2 and 3 are positive *S. aureus*; Lanes 4, 5 and 6 are negative *S. aureus*; Lanes 7 and 8 are negative and positive controls of *S. aureus*, respectively. (**C**) Sequence alignments of the partial *Nuc* gene. Upper line is an annotated *Nuc* gene sequence (NCBI: NC_002758), and lower line is *Nuc* sequence amplified from one of our positive samples. Identity = 93.65%.

Bovine mastitis caused by *S. aureus* is usually asymptomatic and shows no apparent changes in milk. Somatic cell count (SCC) or log-transformed SCC (somatic cell score, SCS) is usually used as an indirect indicator for bovine mastitis susceptibility to responses to the degree of mastitis morbidity^25^,^26^. Therefore, the candidate samples were first selected based on SCS records and *S. aureus* identification. Six Holstein cows were selected from 17 candidate cows based on the SCCs of three consecutive months (Fig. 1, Supplementary Table S1) and milk bacteria identification (Fig. 2).

As shown in Fig. 1 and Table 1, the selected samples were divided into two groups. The SA group (*n* = 3) was naturally infected by *S. aureus* (S1, S2 and S3), whereas the CK group (*n* = 3) was not infected by *S. aureus* (C1, C2 and C3). As expected, the SCS of the SA group was significantly higher than that of the CK group (*P* < 0.01). Moreover, the culture plate of the suspected *S. aureus* positive samples exhibited numerous single colonies after bacterial culture for 24 h and Gram stain was positive, which was checked by *S. aureus*-specific blood plate and Baird–Parker agar (Fig. 2A). By contrast, there was no evidence of the presence of *S. aureus* in healthy controls. These six samples were confirmed by specific molecular identification of *S. aureus*, including PCR amplification (Fig. 2B) and sequencing of the *Nuc* gene (Fig. 2C), which is a representative gene of *S. aureus*. These results indicate that the *S. aureus*-infected cows and control animals selected from one population (Table 1) could be used for subsequent analyses.

### Global DNA methylation profiles of the bovine peripheral blood lymphocytes

**Figure 3:**
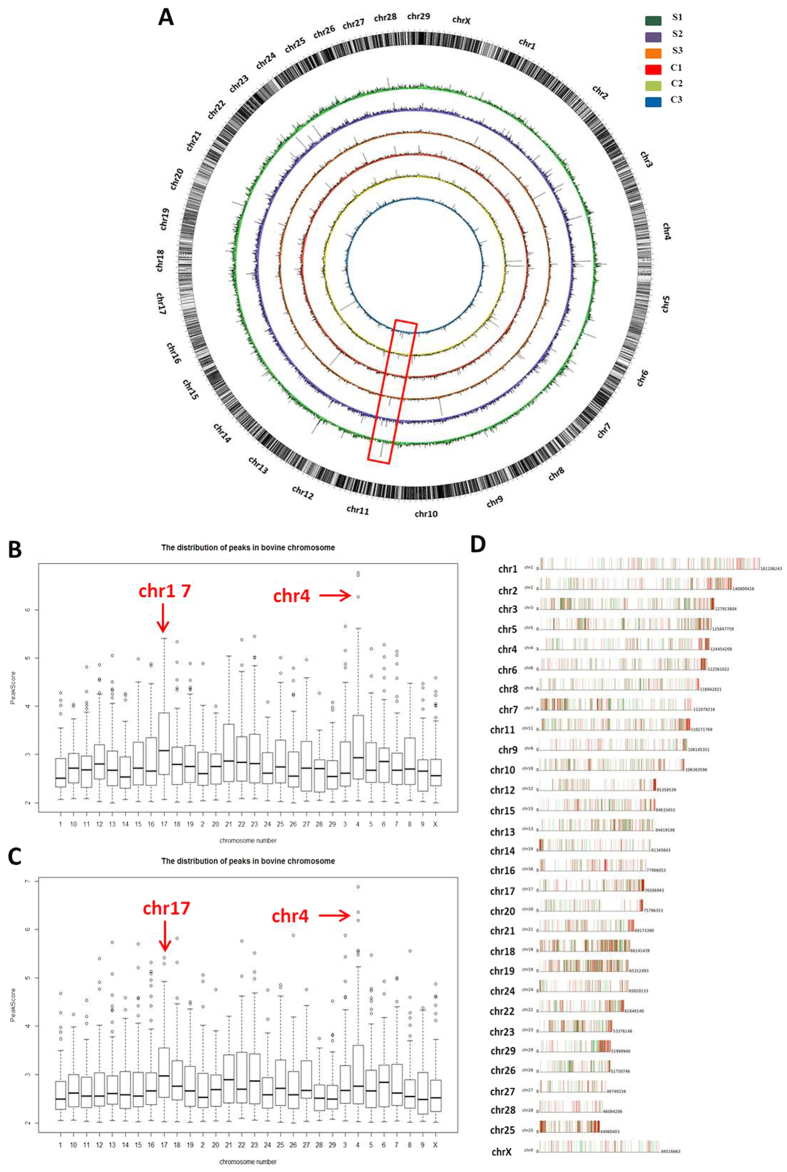
DNA methylation profiles of bovine peripheral blood lymphocytes. (**A**) Global DNA methylation patterns of cows with *S. aureus* subclinical mastitis (the outer three circles) and healthy cows (the inner three circles). Red box shows the differential methylation region between the SA and CK cows. (**B**) Distribution of DNA methylation peaks on each chromosome in the SA group. (**C**) Distribution of DNA methylation peaks in the CK group. Red arrows indicate chromosomes 4 and 17. (**D**) Distribution of differentially methylated peaks on each chromosome in the contrast of SA group and CK group. Red and green bars represent up-methylated and down-methylated peaks, respectively.

Genome-wide DNA methylation levels of peripheral blood lymphocytes of the six cows were analysed by methylated DNA immunoprecipitation combined with microarray (MeDIP-chip) assay. To show the global DNA methylation profiles, the whole genome-wide DNA methylation maps of the six samples were represented by a Circos histogram (Fig. 3A). The DNA methylation levels between the two groups indicated some differences; the methylation levels of cows with *S. aureus* subclinical mastitis were significantly higher than those of healthy cows in BTA11 (bovine chromosome 11, indicated by the red box in Fig. 3A). The distributions of methylation enrichment peaks (EPs) at each chromosome of the SA and CK cows are shown in Fig. 3B,C, respectively. The methylation EPs showed heterogeneous distribution in bovine chromosomes of the two groups. Methylation enrichment of BTA4 and BTA17 was higher compared with other chromosomes in both groups.

Moreover, the differentially methylated peaks were highly distributed on BTA3, BTA18 and BTA19 in the contrast of SA group and CK group (Fig. 3D, Table 2), whereas BTA20 contained a relatively larger gap. The up-methylated peaks were distributed in almost all of the terminals, except chromosomes 1, 14, 16, 24 and X, which might be responsible for the epigenetic regulation of the subtelomere region in cows with *S. aureus* subclinical mastitis.

### DNA methylation variations between the SA and CK cows

To assess overall DNA methylation variations in the genome of cows with *S. aureus* subclinical mastitis and healthy controls, the global DNA methylated peaks in blood lymphocytes of the two groups were obtained. A total of 58,198 methylated peaks were detected in the two groups, in which 29,344 (50.4%) methylated peak numbers were determined in the *SA* group and 28,854 (49.6%) methylated peak numbers were noted in the CK group (Supplementary Table S2).

**Figure 4:**
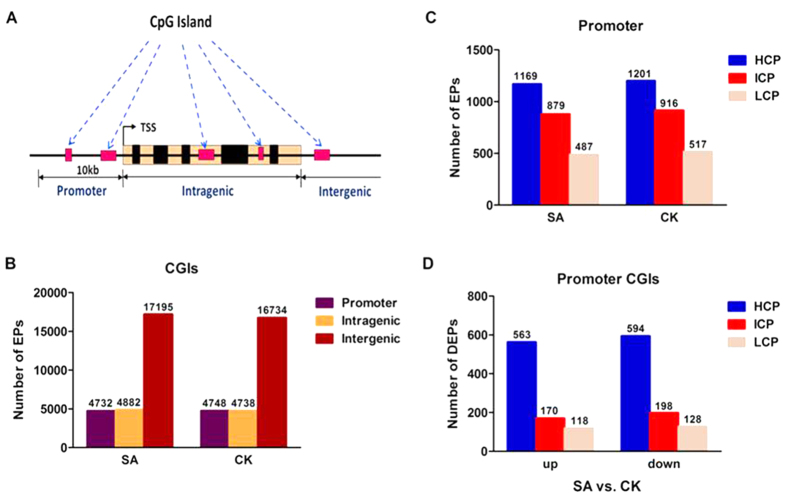
Distribution of DNA methylation enrichment peaks in the genome of SA and CK cows. (**A**) CpG islands (CGIs) and gene transcript regions. TSS: transcriptional start site. (**B**) Number of methylation enrichment peaks (EPs) of different CGI regions in SA and CK cows. (**C**) EP number of three types of promoter regions in the two groups. (**D**) Number of differentially methylated enrichment peaks (DEPs) of the promoter CGI regions in the contrast of SA group and CK group.

Many CpG islands (CGIs) are present in the bovine genome. CGI is defined as the region with a high frequency of CpG sites. In the present study, the bovine CGIs were grouped into three classes according to their distance to the RefSeq genes: promoter CGIs (from about −10 kb to transcriptional start site (TSS)), intragenic CGIs (from the TSS to the transcription terminal site (TTS) within a gene) and intergenic CGIs (the remainder that did not fall into either promoter or intragenic)^27^ as presented in Fig. 4A. The total number of methylation EPs in the three types of CGIs is listed in Fig. 4B. Most of the methylated peaks were distributed in the intergenic CGIs in the cattle genome regardless of SA or CK cows (the brown bars in Fig. 4B) compared with the intragenic or promoter CGIs. These results were consistent with previous studies^19^,^28^.

A promoter is a DNA region that initiates gene transcription. Mammalian promoter regions are normally divided into three types based on their CpG ratio, GC content and length of CpG-rich region: high CpG density promoter (HCP), low CpG density promoter (LCP) and intermediate CpG density promoter (ICP). In the bovine promoter regions (Fig. 4C), we found that the methylated peaks of the HCP type were the highest in both cattle groups compared with those of the ICP and LCP types. In addition, the methylated peaks of SA cows were 32, 37 and 30 less than those of CK cows in the HCP, ICP and LCP types, respectively. Concerning the promoters with CGIs (Fig. 4D), we found that the number of differential methylation enrichment peaks (DEPs) of the HCP type was higher than those of the ICP and LCP types regardless of up- or down-methylated promoters. The number of down-methylated promoters in the comparison of SA versus CK cows was 31, 28 and 10 larger than that of up-methylated HCP, ICP and LCP, respectively.

**Figure 5:**
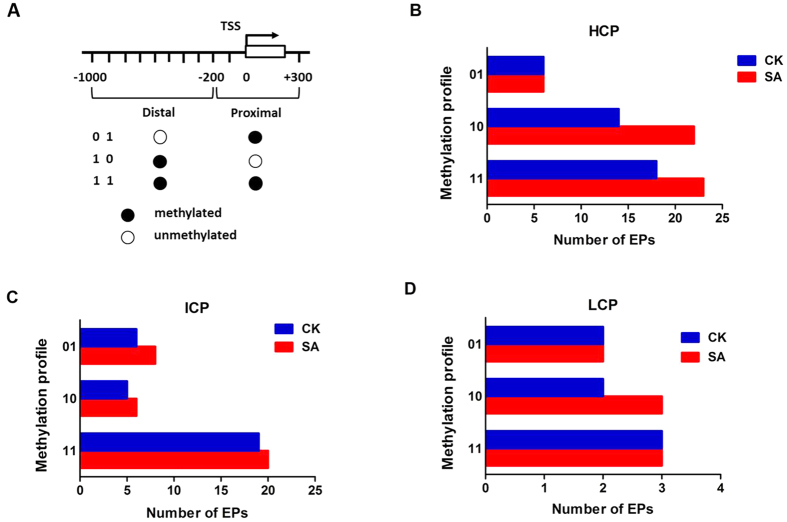
Distribution of different methylation patterns of promoter regions around the TSS in the SA and CK cows. (**A**) Two promoter regions relative to the TSS. Each region was marked as methylated (1) or unmethylated (0). (**B**) Methylated enrichment peak number of HCP (high CpG density promoter) for the SA and CK groups and for three methylation profiles (01: proximally methylated, 10: distally methylated and 11: fully methylated). (**C,D**) were the same as (**B**) for ICP (intermediate CpG density promoter) and LCP (Low CpG density promoter), respectively.

The key promoter region is defined as the −1000 bp to +300 bp regions around the TSS of genes. To thoroughly identify DNA methylation patterns in bovine promoter regions, we classified promoter regions into proximal (−200 bp to +300 bp) and distal (−200 bp to −1000 bp) regions relative to TSS, as presented in Fig. 5A. The proximal and distal regions were then defined as methylated (1) or unmethylated (0), respectively. Thus, the promoter regions were divided into three patterns according to their methylation profiles, as shown in Fig. 5A 18. The number of methylated EPs with specific methylation patterns in the promoter regions in the SA and CK groups is shown in Fig. 5B–D. In general, most HCP were distally methylated (‘10’ pattern) and fully methylated (‘11’ pattern) (Fig. 5B), whereas most ICP were fully methylated (‘11’ pattern) (Fig. 5C). Based on the classification of the promoters, the data indicated that the promoter types HCP, ICP and LCP in the SA group were hypermethylated compared with those in the CK group, except the ‘01’ pattern in HCP and LCP as well as “11” pattern in LCP.

### Differentially methylated genes in the SA and CK cows

**Figure 6:**
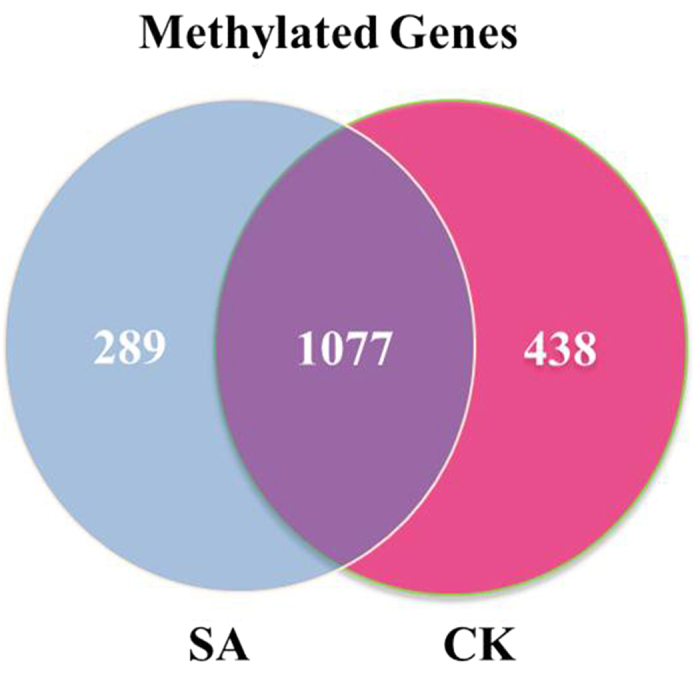
Methylated genes that were unique or shared between the SA and CK groups.

DNA methylation can induce aberrant promoter function of a gene. In the present study, 2881 methylated genes were detected, among which 1366 and 1515 methylated genes were found in the SA and CK cows, respectively, whereas 1077 genes were shared in the two groups (Fig. 6). To identify the differentially methylated genes, the methylated peaks between the SA and CK groups were compared. Consequently, a total of 1078 differentially methylated genes were observed (*P* ≤ 0.001, Supplementary Table S3). Of these genes, 527 genes were up-methylated and 551 genes were down-methylated. These results suggest that the number of differentially up-methylated genes was moderately decreased in the contrast of SA group and CK group compared with the number of down-methylated genes.

**Figure 7:**
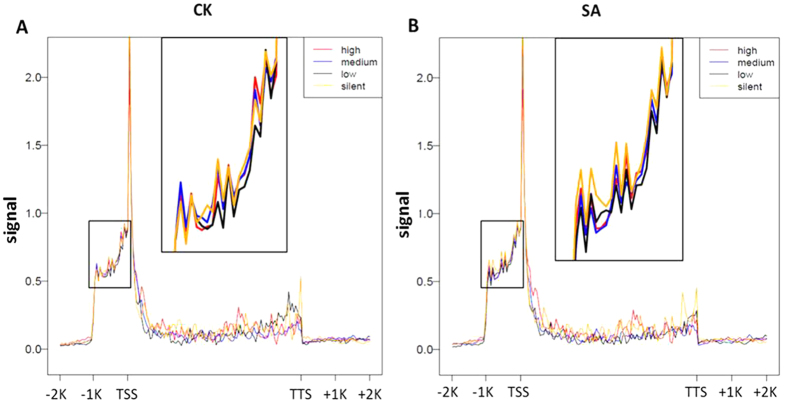
DNA methylation profiles were compared across four gene sets in CK (**A**) and SA (**B**) cows. The X-axis shows different gene regions, whereas the Y-axis indicates the normalised MeDIP-chip signal. Genes were classified into four sets according to their expression levels as follows: silent, low expression, medium expression and high expression. Each gene set included 180 genes. Black box indicates the magnified −1 K region of the TSS.

The gene promoter region near the TSS is critical for regulating gene expression^15^. To identify how DNA methylation regulates gene expression, we compared DNA methylation levels across four gene sets corresponding to four gene expression levels (silent, low expression, medium expression and high expression) in the upstream 2 kb region of the TSS, gene body and downstream 2 kb region of the TTS (Fig. 7). The DNA methylation levels of highly expressed genes and silent genes showed a clearly negative correlation with the gene expression levels across the whole upstream 1 kb region of the TSS in SA cows (Fig. 7B), whereas the trend was not evident in CK cows (Fig. 7A). The relationship between DNA methylation levels and transcriptional expression was not found in the gene body and downstream of the TTS in the two groups. These results indicate that transcriptional repression driven by DNA methylation mainly occurred from −1 K to TSS, which might contribute to the progress of subclinical mastitis induced by *S. aureus* in dairy cattle.

### Functionally relevant genes regulated by DNA methylation in cows with *S. aureus* subclinical mastitis

To identify the functional genes repressed by DNA methylation in cows with *S. aureus* subclinical mastitis, we further compared the DNA methylation profiles and gene expression data in SA versus CK. Differentially methylated genes in the promoter regions were selected to investigate concomitant expression changes in SA versus CK cows. We obtained a total of 705 differentially expressed genes by digital gene expression (DGE) (false discovery rate (FDR) <0.2, Supplementary Table S4). Combined with 1078 differentially methylated genes and using the screening criteria chosen (FDR < 0.2 and −log_10_ *P* ≥ 3), 58 genes were shared between differentially expressed genes and differentially methylated genes (Supplementary Fig. S1, Supplementary Table S5). Of these 58 genes, 12 were identified as hypermethylated and down-regulated (Table 3), and eight were hypomethylated and up-regulated (Table 4). The results suggested more hypermethylated and down-regulated genes in peripheral blood lymphocytes of cows with *S. aureus* mastitis compared with those of healthy cows.

**Figure 8:**
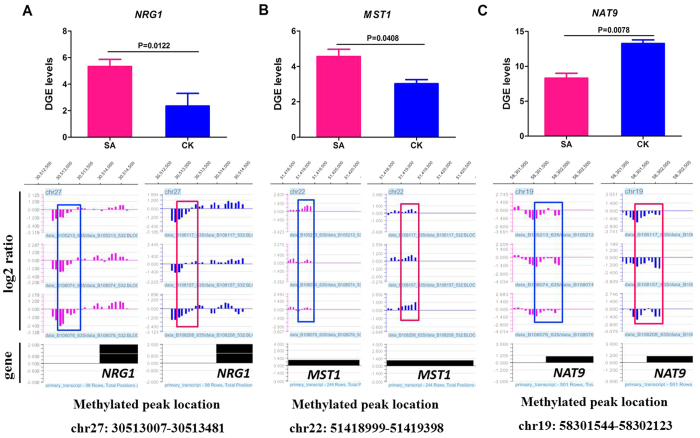
Transcriptional levels (upper panel) and methylated peaks (lower panel) of three differentially methylated and expressed genes. (**A**) *NRG1* gene (relatively hypomethylated and up-regulated in SA cows). (**B**) *MST1* gene (relatively hypomethylated and up-regulated in SA cows). (**C**) *NAT9* gene (relatively hypermethylated and down-regulated in SA cows). Red and blue boxes indicate the differentially methylated peaks in SA and CK cows, respectively. The log_2_ ratio represents the logarithm of the ratio of MeDIP DNA probe signal value and input DNA probe signal value for individuals, and it is normalised. Black solid box indicates the transcription region of a gene. *P* < 0.01 and *P* < 0.05 indicate highly significant difference and significant difference between the SA group and CK group, respectively.

The transcriptional levels and methylated peaks of three inflammation-related genes (*NRG1, MST1* and *NAT9*) are shown in Fig. 8, which revealed a negative correlation with statistical significance of *P* < 0.05. Thus, these genes could be powerful candidate methylation target genes related to susceptibility or resistance to bovine *S. aureus* subclinical mastitis.

**Figure 9:**
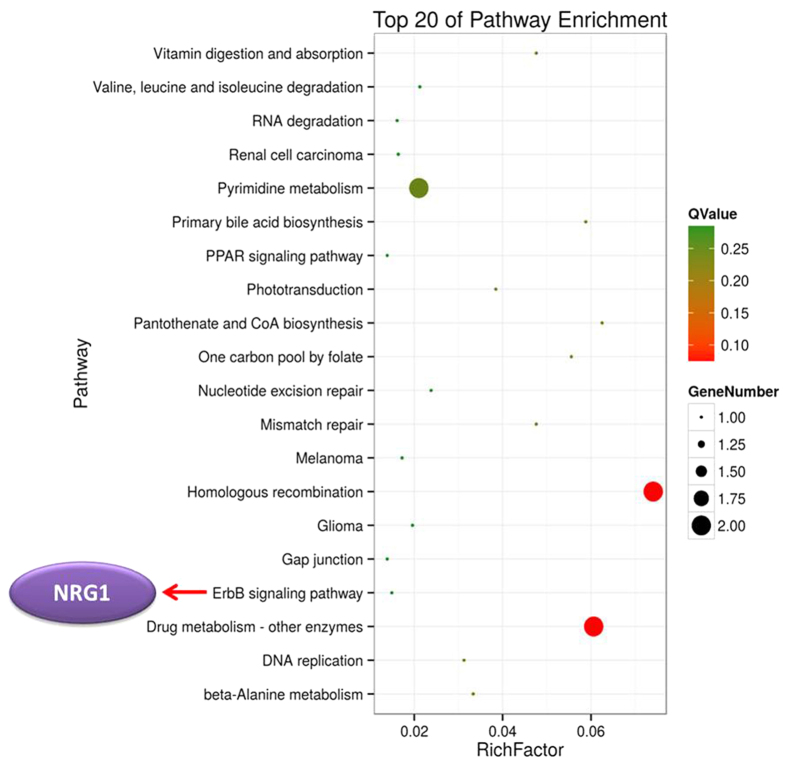
Scatter plot of the top 20 KEGG enrichments. The X-axis represents the rich factor. The rich factor is the ratio of differentially methylated and expressed gene numbers annotated in this pathway term to all gene numbers annotated in this pathway term. The Y-axis is the pathway enrichment terms. Q-value represents the corrected *P*, and a small Q-value indicates high significance. The red arrow indicates the functional related gene within the pathway.

To investigate the biological processes and functional pathways, we further performed gene ontology (GO) term and pathway enrichment analyses for 58 differentially methylated and expressed genes. GO analysis showed that some genes were involved in response to biological process and transcription regulator activity, although no statistical significance was noted (Supplementary Fig. S2). Pathway enrichment analysis identified 34 pathways, and the top 20 enriched pathways are shown in Fig. 9. Pathway enrichment analysis revealed that the genes regulated by methylation were associated with inflammation and cancer progression, such as the ErbB signalling pathway (Q-value = 0.29), systemic lupus erythematosus (Q-value = 0.24) and melanoma (Q-value = 0.29) (Fig. 9). Notably, the *NRG1* gene was observed in the ErbB signalling pathway.

### Validation of MeDIP-chip data by bisulphite sequencing PCR (BSP)

**Figure 10:**
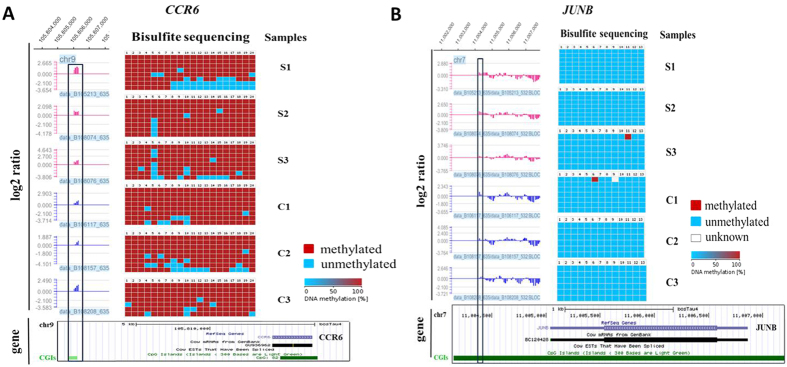
Bisulphite sequencing PCR. The log_2_ ratio of DNA methylation enrichment was given for the 5′ end of two genes in peripheral blood lymphocytes of the SA group (S1, S2 and S3) and CK group (C1, C2 and C3). Bisulphite cloning and sequencing results were shown with columns representing CpG positions and rows representing individual clones. Blue shows unmethylated CpG sites, whereas red indicates methylated CpG sites. Black box indicates the methylated peak region in SA and CK. The log_2_ ratio represents the logarithm of the ratio of MeDIP DNA probe signal value and input DNA probe signal value for individuals, and it is normalised. The box below indicates the gene structure in UCSC. (**A**) *CCR6*: high methylated peak enrichment; (**B**) *JUNB*: nearly unmethylated peak enrichment.

To assess the accuracy of the MeDIP-chip mapping results, the *CCR6*, *JUNB* and *HOXA6* genes were selected to validate promoter DNA methylation enrichment using BSP. The *CCR6*, *JUNB* and *HOXA6* genes were arbitrarily chosen from highly methylated, unmethylated and lowly methylated genes, respectively. In general, we found good coherence between MeDIP-chip results and BSP results, *i.e. CCR6* exhibited hypermethylated enrichment (Fig. 10A), *JUNB* showed nearly unmethylated enrichment (Fig. 10B) and *HOXA6* demonstrated hypomethylated enrichment in the six samples (Fig. S3).

## Discussion

Bovine subclinical mastitis induced by *S. aureus* is a serious concern in the dairy industry and public health. This study generated a genome-wide DNA methylation profile of bovine *S. aureus* subclinical mastitis and identified significant DNA methylation markers, as well as their targeted novel candidate genes relevant to the responses of dairy cows to *S. aureus*.

Bovine mastitis induced by *S. aureus* is usually asymptomatic and can be an invisible reservoir of *S. aureus*. It may threaten other cows in the same population and even human health through milk products. The SCC in bovine milk and its log-transformed score (SCS) have relatively higher heritability compared with mastitis, so the SCC and SCS are widely used as indicators for mastitis management and prevention^25^,^26^,^29^. In general, the SCC of healthy cows is normally less than 100,000 cells/mL, whereas that of subclinical or clinical mastitis cows is higher, usually more than 300,000 cells/mL^30^,^31^. To exactly detect *S. aureus* subclinical mastitis in cows, we proposed that cows with high SCC (>300,000 cells/mL) for three consecutive months could be candidates of bovine *S. aureus* subclinical mastitis. However, considering that the SCC of 40% cows infected by *S. aureus* was lower than 200,000 cells/mL^32^, molecular identification of *S. aureus* was further conducted for the candidate cows. The SCC and *S. aureus* identification were critical for subsequent comparisons of SA and control cows for genome-wide DNA methylation regulation analysis.

MeDIP-chip is a widely used assay to generate comparative genome-wide DNA methylation dynamic patterns on mammalian tissues and cell samples^33^. In the present study, we generated the first global DNA methylation profiles of peripheral blood lymphocytes in cows with *S. aureus* subclinical mastitis compared with healthy controls using MeDIP-chip. We found that the total DNA methylation levels were higher in cows with *S. aureus* mastitis than those in control cows; these high levels might be responsible for high methylation peaks in the intergenic CGIs of the SA group in the cattle genome. The DNA methylation level dramatically peaked at the TSS, sharply dropped and plateaued after the TTS. The DNA methylation level of peripheral blood lymphocytes in cows with *S. aureus* mastitis showed analogous tendency to bovine placentas, muscle tissues and other mammals^19^,^21^,^28^,^34^,^35^. However, the DNA methylation level sharply decreased after the TSS towards the gene body region; this finding was inconsistent with bovine placentas and muscle tissue^19^,^28^. We infer that the relatively low methylation level of the gene body in bovine peripheral blood lymphocytes may be correlated with cell- and tissue-specific transcriptional programs and play a different role in regulating gene expression. Besides, the 385,000 probes on bovine MeDIP-chip (Roche–NimbleGen) were designed based on CGIs and gene promoters, thus the chip information about gene body regions might be insufficient. Thus, MeDIP-seq or whole-genome bisulphite sequence will be able to shed light on this point in cows with *S. aureus*-infected subclinical mastitis.

The chromosome terminal is a specific structure that consists of telomere (repetitive sequence) and subtelomeric (repetitive DNA includes a small quantity of genes) regions^36^,^37^. In normal somatic cells, telomere shortening shortens the human lifespan and contributes to the development of age-related pathologies^38^. By contrast, most tumour cells aberrantly elevate telomerase levels, maintain telomeres and divide indefinitely^39^. Telomeric DNA repeat sequences cannot be methylated, but the subtelomere region is heavily methylated and enriched with CGIs and histone markers^36^. Several studies have implicated that the hypomethylation of the subtelomere region can increase telomere recombination and facilitate telomere elongation in human cancer cells^40^. In the present study, the up-methylated peaks were widely distributed in the terminal of the bovine chromosome in SA group versus CK group. We speculate that the peripheral blood lymphocytes of *S. aureus*-infected cows could be affected by the hypermethylation of the chromosome terminal region.

Most of the differentially methylated regions were enriched at the gene promoter^41^. Previous reports revealed that DNA methylation and transcriptional repression in ICP have a stronger correlation compared with that in HCP and LCP^42^. We also analysed the DNA methylation patterns in the promoter regions for 14871 bovine genes (385,000 probes on bovine MeDIP-chip). In general, the methylation level of the promoter region was significantly lower than that of the intergenic regions in bovine blood lymphocytes. The methylation level of HCP was higher than that of ICP and LCP (Fig. 4C). These findings are consistent with the findings of previous research^19^,^28^. Besides, the number of down-methylated CGI promoters was higher than that of up-methylated HCP, ICP and LCP in SA versus CK (Fig. 4D). The result suggests that *S. aureus* might induce moderately down-methylated CGI promoters in the bovine genome. Notably, the numbers of enriched methylation peaks around the TSS in cows with *S. aureus* subclinical mastitis were moderately higher compared with those in healthy cows (Fig. 5). Moreover, DNA methylation of the proximal and distal promoter regions of HCP and ICP in cows with *S. aureus* subclinical mastitis was higher than that in control cows. Taken together, these results suggest that promoter hypermethylation in SA cows might be correlated with the pathogenesis of subclinical mastitis induced by *S. aureus*.

Promoter methylation is related to transcriptional repression^18^. By integrating DNA methylation data and gene expression data, we found that promoter methylation was negatively correlated with gene expression in cows with *S. aureus* mastitis. The low expressed genes displayed relatively high DNA methylation in the 1 kb region upstream of TSS, whereas highly expressed genes displayed relatively low DNA methylation. Consistent with previous reports on humans, cattle and chicken^19^,^28^,^35^,^43^, DNA methylation levels around the TSS are repressive epigenetic markers that down-regulate gene expression. However, we did not observe transcription repression of promoter methylation in healthy cows. Emerging evidence suggested that extremely hypermethylated promoter regions were observed in the tumour cells compared to the healthy cells^18^,^44^,^45^. Moreover, hypermethylation in the promoter induced by bacterial infection has been identified in mice^46^. Thus, we speculated that *S. aureus* may contribute to promoter hypermethylation and transcriptional repression in *S. aureus*-infected mastitis cows.

Pathway analysis revealed 58 differentially methylated and expressed genes involved in several key functional pathways, including homologous recombination and mismatch repair, one carbon pool by folate and the ErbB signalling pathway, which may be closely related to progression of bovine *S. aureus* mastitis. Among these genes, 35% differentially DNA methylated promoter regions were negatively correlated with gene expression, and a positive correlation (27% hypermethylated and up-regulated genes, 38% hypomethylated and down-regulated genes) was also detected in the present study. This phenomenon was also noted in human lung adenocarcinoma^45^. These results suggest that dynamic DNA methylation regulation occurred in the blood lymphocytes of bovine subclinical mastitis induced by *S. aureus*.

The targeted genes modified by differential DNA methylation in SA group versus CK group should be paid more attention. *NRG1* (neuregulin 1) was hypomethylated and up-regulated in SA cows, and it was associated with the ErbB signalling pathway. A previous study proved that overexpression of the NRG1/ErbB system is associated with tumourigenesis and cardiovascular function disease^47^,^48^. Notably, the functions of the *NAT9* (N-acetyltransferase 9) and *MST1* (macrophage stimulating 1) genes were closely relevant to inflammation responses, although they were not significantly enriched in any pathway. *NAT9*, a hypermethylated and down-expressed gene in SA cows, encodes a new member of the *N*-acetyltransferase superfamily and has been reported to be a susceptibility factor for psoriasis, which is a chronic inflammatory skin disorder disease^49^. *MST1*, a hypomethylated and up-regulated gene in the SA group, is also known as *MSP* (macrophage-stimulating protein), and it is involved in the Msp/Ron receptor signalling pathway^50^,^51^. The Msp/Ron signalling pathway has been proven to regulate mononuclear phagocytes and ciliary motility, and it might participate in the host defense^52^. The repression of the *MSP/MST-1* gene might contribute to oncogenesis^53^. Considering the functions of these genes, we believe that *NRG1*, *MST1* and *NAT9* are strongly correlated with the progression of *S. aureus* subclinical mastitis, as evidenced by their apparent expression changes in bovine mammary epithelial cell lines (Mac-T cells) after *S. aureus* challenge (Fig. S4).

Many immune-related genes are differentially regulated in bovine subclinical mastitis infected by *S. aureus*. At the early hours of co-culture between bovine mammary epithelial cells (bMECs) and *S. aureus*, interleukin-8 (*IL-8*), tumour necrosis factor alpha (*TNF-α*) and interleukin-1*β* (*IL-1β*) were highly expressed in bMECs infected with *S. aureus* than in uninfected bMECs^54^. In addition, protein levels of serum amyloid A3, cathelicidin 4 and complement component 3 (C3) in milk whey samples from naturally infected cows with *S. aureus* were differentially expressed between *S. aureus* mastitis samples and normal samples^55^. Although these genes and proteins were involved in host defence against *S. aureus*, we did not find evidence of DNA methylation changes in these genes or the encoding genes in the present study, which might be modified by other epigenetic modifications and warrant further exploration.

In summary, the DNA methylation profiles of peripheral blood lymphocytes in cows with *S. aureus* mastitis and healthy controls, as well as integrative analysis of DNA methylation and transcriptome, accomplished three goals: (1) the first genome-wide DNA methylation patterns of bovine peripheral blood lymphocytes were generated; (2) the identified functional DNA methylation changes were related to the expression of genes involved in the bovine inflammatory response and resistance to *S. aureus* mastitis; and (3) the novel bovine mastitis-specific genes (specifically *MST1*, *NRG1* and *NAT9*) targeted by DNA methylation changes could be pursued as potential biomarkers for the prevention of *S. aureus* subclinical mastitis. Our studies lay the groundwork for epigenetic modification and mechanistic studies on the resistance and prevention of bovine *S. aureus* subclinical mastitis.

## Materials and Methods

### Animals and sampling

All protocols for the collection of blood and milk samples of experimental cows were reviewed and approved by the Institutional Animal Care and Use Committee at China Agricultural University. The experiment was conducted according to regulations and guidelines established by this committee. All efforts were made to minimise suffering.

A total of 17 Holstein cows were selected from a dairy herd in Beijing Suburb, China, based on their performance testing data (DHI) records throughout the whole year. The DHI data were provided by the official Dairy Data Centre of China (Beijing, China). About 100 mL of fresh milk used for bacteria identification was aseptically collected from all lactating quarters and equally mixed for each cattle^56^. Simultaneously, 40 mL of blood samples used for separating peripheral blood lymphocytes was obtained from the jugular vein for each animal. Peripheral blood lymphocytes were prepared by Lymphocyte Separation Medium (TBDsciences, Tianjin, China) according to the manufacturer’s instructions, and the purity was 90–95%.

### *S. aureus* isolation and identification

Bacteriological culture of milk samples was carried out according to National Mastitis Council standards. Firstly, 100 μL of milk was transferred into a blood agar plate and spread with a glass spreader. Subsequently, the agars were incubated at 37 °C for 24 h^57^. After blood plate culture of milk samples, suspected colonies (with clear zones of haemolysis) of *S. aureus* were purified in Baird–Parker agar (specific to *S. aureus*) culture medium (Beijing Land Bridged Technology Ltd., Beijing, China) and cultured at 37 °C for 24 h. Typical *S. aureus* colonies on Baird–Parker agar culture medium were identified by Gram stain. Finally, bacteria that tested positive in Gram stain were *S. aureus*.

To further confirm the presence of *S. aureus* in milk, molecular methods were simultaneously developed according to a modified protocol^58^. Initially, 650 μL of milk sample was diluted with 650 μL of NaCl (0.9%) and then centrifuged for 15 min at 8,000 rpm at 4 °C. Secondly, 700 μL of NaCl (0.9%) was used to suspend the precipitate, which was centrifuged for 15 min at 8,000 rpm at 4 °C. Thirdly, 350 μL of extraction solution and 250 μL of binding solution were added to the tube; the cells were then mixed by inverting the tube for 5 min. The mixture was centrifuged for 2 min at 5,500 rpm at 4 °C. Fourthly, 300 μL of extraction solution was added to the tube for resuspending the cell pellet. The samples were centrifuged for 2 min at 5,500 rpm at 4 °C. Fifthly, 600 μL of washing solution was used to resuspend the pellets. The samples were centrifuged for 2 min at 5,000 rpm at 4 °C. Sixthly, the pellets were washed in 500 μL of absolute ethanol solution. The samples were centrifuged for 3 min at 6,000 rpm at 4 °C, and the supernatant was removed. The pellets were dried in an oven at 50 °C. Finally, 60–100 μL of elution buffer preheated at 75 °C was used to resuspend the pellet, and the resultant solution was incubated for 15 min at 75 °C. The samples were centrifuged for 5 min at 6,000 rpm at 4 °C, and the supernatant was transferred to a new tube. The DNA template was stored at −20 °C for PCR.

The PCR assay was performed for amplifying the *Nuc* gene according to a previous protocol^59^. The primer sequence used for PCR is listed in Supplementary Table S6. The PCR reaction was performed in 25 μL, containing 3 μL of genomic DNA, 1 μL of each primer (10 μmoL), 12.5 μL of Taq^TM^ Premix and 7.5 μL of ddH_2_O. PCR was performed using the following program: 94 °C for 10 min; 35 cycles of 94 °C for 30 s, 59 °C for 30 s and 72 °C for 30 s; and 72 °C for 7 min. Finally, six Holstein cows were selected from 17 Holstein cows, of which three cows infected with *S. aureus* were grouped into the SA group, and the other three without *S. aureus* infection comprised the CK group.

### MeDIP-chip

Genomic DNA was isolated from peripheral blood lymphocytes of six cows using a Wizard Genomic DNA Purification Kit (Promega, Shanghai, China). The purified DNA was then quantified and quality assessed by Nanodrop ND-2000. Genomic DNA was sonicated to generate 200–1000 bp fragments. Immunoprecipitation of methylated DNA was performed using Biomag^TM^ magnetic beads coupled to mouse monoclonal antibody (Diagenode) against 5-methylcytidine. The immunoprecipitated DNA was eluted and purified by phenol chloroform extraction and ethanol precipitation. The total input genomic DNA and immunoprecipitated DNA were labelled with Cy3- and Cy5-fluorophere, respectively, and hybridised to custom-designed NimbleGen Cow CpG island plus Ensemble promoter arrays (NimbleGen Systems Inc., Madison, USA). The CpG array covered all known CGIs annotated by UCSC and all well-characterised RefSeq promoter regions from about −1000 bp to +300 bp of the TSSs, which completely covered ~385,000 probes. Scanning was performed with the Axon GenePix 4000B microarray scanner following the manufacturer’s guidelines detailed in the NimbleGen MeDIP-Chip protocol (NimbleGen Systems Inc., Madison, USA).

### Normalisation and analysis of MeDIP-chip data

The DNA methylation level was represented by the log_2_ ratio value (represented enrichment intensity of each probe mapped to the gene promoter and CGIs between MeDIP DNA and input DNA) and *P* value. The formula used to calculate the log_2_ ratio is as follows: log_2_ ratio = log_2_ (the fluorescence signal of MeDIP DNA/the fluorescence signal of input DNA). To avoid technical variability and evaluate methylation differences between samples, the log_2_ ratio obtained from raw data values should be normalised. We performed median centering, quantile normalisation and linear smoothing using Bioconductor packages Ringo, Limma and MEDME.

From the normalised log_2_ ratio data, a sliding-window (750 bp) peak-finding algorithm provided by NimbleScan v2.5 (Roche-NimbleGen) was applied to analyse the MeDIP-chip data. A one-sided Kolmogorov–Smirnov (KS) test was applied to determine whether the probes are drawn from a significantly more positive distribution of intensity log_2_ ratio than those in the rest of the array. Each probe received a −log_10_ *P* score from the windowed KS test around that probe. If several adjacent probes rose significantly above a set threshold, the region was assigned to an EP. NimbleScan detects peaks by searching for at least two probes above a *P*-value minimum cut-off (−log_10_). Peaks within 500 bp of each other are merged. The significance threshold of *P* in multiple tests was set based on the FDR. After multiple test correction, we used *P* ≤ 0.001 and |log_2_ ratio| ≥ 1 as the threshold to assess the significance of differentially methylated genes.

### DGE and analysis

Total RNA from peripheral blood lymphocytes was extracted from six samples (three *S. aureus* mastitis cows and three healthy cows) with mirVana miRNA Kit (Ambion, Austin, USA). Approximately 6 mg of total RNA was transcribed into double-stranded cDNA through a Reverse Transcription Kit (Applied Biosystems, Waltham, USA). The protocols of DGE-seq were conducted as previously described^60^. Raw data from DGE-seq were mapped to the bovine reference genome by SOAP 2.21 software, and all genes were annotated by Ensembl BioMart. Raw sequences were transformed into clean tags after data processing. All clean tags were mapped to the reference sequences, and only tags with perfect matching or 1 bp mismatch were considered.

The expression level of one gene was represented by TPM (number of transcript copies in per million clean tags), which was equal to the copy number of clean tags for this gene divided by the total number of clean tags and multiplied by one million^61^. The DEGs between SA group and CK group were analyzed based on Poisson distribution according to previous study in which the algorithm was described in detail^62^. The corresponding *P* values of DEGs were calculated based on normalised expression. The significant threshold of *P* in multiple tests was set based on FDR. The fold changes (log_2_ ratio) were also estimated based on the normalised gene expression level in each sample. The differentially expressed genes were selected based on the expression profiles and the following criteria: the change in gene expression levels in SA versus CK was |log_2_ ratio| ≥ 1.5 and FDR ≤ 0.2.

### BSP

To confirm the reliability of MeDIP-chip data, the methylation level of three genes (*JUNB*, *CCR6* and *HOXA6*) was identified by BSP. Genomic DNA (1000 ng) from the SA and CK cows was treated with bisulphite sodium using the EZ DNA Methylation Kit (Zymo Research, Irvine, USA). The bisulphite-treated DNA was used for PCR. PCR primers were designed by online MethPrimer software^63^ (Supplementary Table S7). The BSP reaction was performed in 25 μL, containing 2 μL of genomic DNA, 1 μL of each primer (10 μmoL), 12.5 μL of Zymo Taq^TM^ Premix and 8.5 μL of ddH_2_O. PCR was performed using the following program: 95 °C for 10 min; 40 cycles of 94 °C for 30 s, 60 °C for 40 s and 72 °C for 45 s; and 72 °C for 7 min. The PCR products were detected by gel electrophoresis and cloned into the pMD19-T vector (TaKaRa, Dalian, China). Seven to ten positive clones for each gene per sample were randomly selected for sequencing (Sangon, Shanghai, China). The final sequence results were processed by online software BISMA^64^.

### Cell culture and bacterial challenge

Mac-T cells (a bovine mammary epithelial cell line) were re-suspended in warm growth medium. DMEM was supplemented with 10% foetal bovine serum and 100 U/mL penicillin and streptomycin (100 mg/mL) (Gibco BRL, New York, USA). Cells were cultured in a 25 cm^2^ tissue culture flask at 37 °C in a 5% CO_2_ humidified incubator. Mac-T cells were cultured up to a maximum of three passages to reduce the risk of aberrant expression caused by extended culturing. The medium was changed once every 24 h. At 85% confluence, the cells were split by adding 1 mL of 0.25% trypsin/EDTA (Gibco BRL, New York, USA) after washing with 2 mL of DPBS (Gibco BRL, New York, USA). The cells were centrifuged in 5 mL of DMEM growth medium for 5 min at 1000 rpm, seeded at a concentration of 5 × 10^5^ cells in a six-well cell culture plate (Corning, New York, USA) and grown in a growth medium at 37 °C in 5% CO_2_ humidified incubator. At 85% confluence, Mac-T cells were stimulated with *S. aureus* (1 × 10^8^ CFU/mL) isolated from subclinical mastitis cows at 6 h to enable a ratio of 10 bacteria to 1 Mac-T cell (MOI = 10:1). Control group cells were treated with the same volume of DMEM growth media for 6 h. Each experimental treatment was conducted in triplicate. After 6 h, the media were removed and cells were washed three times in DPBS, harvested in 1 mL of Trizol (Invitrogen, Carlsbad, USA) and stored at −80 °C until RNA extraction.

### Real-time quantitative PCR (qPCR)

For validation of inflammation-related genes in bovine subclinical mastitis, the mRNA expression levels of three inflammation- and disease-related genes (*NRG1*, *MST1* and *NAT9*) were identified by qPCR in bovine mammary epithelial cells (Mac-T cells). Trizol method was used for RNA extraction from six samples of Mac-T cells (three *S. aureus* treated and three untreated control Mac-T cells). cDNA was synthesised using the PrimeScript^TM^ RT reagent kit (Takara, Dalian, China). qPCR was performed using SYBR Green I Master kit (Roche Diagnostics GmbH, Mannheim, Germany) on the LightCycler® 480 II (Roche Diagnostics Ltd., Basel, Switzerland) according to the manufacturer’s instructions. Bovine *GAPDH*, *β-actin* and *18S rRNA* were used as reference genes to normalise to the geometric mean of data from target genes by NormFinder software packages^65^. The primer sequences used for qPCR are listed in Supplementary Table S6. Each RNA sample was analysed in triplicate. The relative mRNA expression levels of the target genes were calculated using the 2^−△△Ct^ method.

### GO annotation and the KEGG pathway

GO enrichment and KEGG pathway analyses were conducted for differentially methylated and expressed genes to investigate their biological processes and functions. GO enrichment and KEGG pathway analyses were carried out using the DAVID Functional Annotation Tool with a 0.05 cut-off for Benjamini adjusted *P* value (Q-value) (http://david.abcc.ncifcrf.gov/). The differentially methylated and expressed genes were classified into cellular component, molecular function and biological process using GO annotation.

## Additional Information

**How to cite this article**: Song, M. *et al*. Combined analysis of DNA methylome and transcriptome reveal novel candidate genes with susceptibility to bovine *Staphylococcus aureus* subclinical mastitis. *Sci. Rep*. **6**, 29390; doi: 10.1038/srep29390 (2016).

## Supplementary Material

Supplementary Information

Supplementary Table S1

Supplementary Table S2

Supplementary Table S3

Supplementary Table S4

Supplementary Table S5

Supplementary Table S6

Supplementary Table S7

## Figures and Tables

**Table 1:** Basic information and bacterial culture of the six Holstein samples.

Group	Sample ID	DIM	Parity	HTM (kg)	SCC × 1000/mL	Bacterium
SA	S1	305	3	28.1	436	*S. aureus*
	S2	283	1	32.67	1106	*S. aureus*
	S3	285	1	35.17	224	*S. aureus*
CK	C1	166	3	42.67	48	–
	C2	133	1	29.33	27	–
	C3	102	1	43.50	104	–

Note: SA - *S. aureus* subclinical mastitis cows; CK - healthy cows; DIM indicates days in milk; HTM indicates herd test milk; SCC indicates somatic cell counts per millilitre of milk sample; – indicates samples without identified bacteria.

**Table 2:** Number of methylated peaks in different chromosomes.

SA vs. CK	Methylated peak (Chromosome, Chr)									
	Chr1	Chr2	Chr3	Chr4	Chr5	Chr6	Chr7	Chr8	Chr9	Chr10
Up	116	145	**174**	113	155	132	156	86	84	111
Down	119	130	**203**	135	175	120	178	108	81	137
	Chr11	Chr12	Chr13	Chr14	Chr15	Chr16	Chr17	**Chr18**	**Chr19**	Chr20
Up	161	88	106	77	108	96	125	**185**	**189**	65
Down	156	79	137	78	104	73	95	**195**	**189**	71
	Chr21	Chr22	Chr23	Chr24	Chr25	Chr26	Chr27	Chr28	Chr29	ChrX
Up	105	111	109	63	172	56	49	45	105	59
Down	94	98	121	65	122	90	48	31	88	79

Note: The chromosome that distributed more methylated peaks is shown in bold.

**Table 3:** Hypermethylated and down-regulated genes in cows with *S. aureus* subclinical mastitis.

Gene ID	WikiGene_name^a^	Chr.	WikiGene_description^b^
ENSBTAG00000015837	*P2RY12*	chr1	Purinergic receptor P2Y12
ENSBTAG00000007190	*THAP6*	chr6	THAP domain containing 6
ENSBTAG00000005115	*SLC31A2*	chr8	Solute carrier family 31 (copper transporters), member 2
ENSBTAG00000003887	*ECHDC1*	chr9	Enoyl-CoA hydratase domain-containing protein 1
ENSBTAG00000017811	*SLC39A9*	chr10	Solute carrier family 39 (zinc transporter), member 9
ENSBTAG00000016312	*LGALS4*	chr18	Galectin-4 (Gal-4)
ENSBTAG00000001782	*MRPS12*	chr18	28S ribosomal protein S12, mitochondrial Precursor (S12mt)(MRP-S12)
ENSBTAG00000023632	*FBXL8*	chr18	F-box/LRR-repeat protein 8 (F-box and leucine-rich repeat protein 8)
ENSBTAG00000017751	*RGS9*	chr19	Regulator of G-protein signalling 9 (RGS9)
**ENSBTAG00000011387**	***NAT9***	**chr19**	**N-acetyltransferase 9**
ENSBTAG00000004635	*LLGL1*	chr19	Lethal giant larvae homolog 1
ENSBTAG00000001615	*RUNDC3A*	chr19	RUN domain-containing protein 3A

Associated gene with inflammation and disease is shown in bold; Chr refers to chromosome.

^a^Gene name.

^b^WikiGene_description from Wikipedia website, https://en.wikipedia.org/wiki/Main_Page.

**Table 4:** Hypomethylated and up-regulated genes in cows with *S. aureus* subclinical mastitis.

Gene ID	WikiGene_name^a^	Chr.	WikiGene_description^b^
ENSBTAG00000021996	*GATAD1*	chr4	GATA zinc finger domain-containing protein 1
ENSBTAG00000012741	*CCPG1*	chr10	CCPG1 protein fragment
ENSBTAG00000016367	*RBM18*	chr11	Probable RNA-binding protein 18 (RNA-binding motif protein 18)
ENSBTAG00000015164	*SLC27A5*	chr18	solute carrier family 27 (fatty acid transporter), member 5
**ENSBTAG00000011585**	***MST1***	**chr22**	**Macrophage stimulating 1 (hepatocyte growth factor-like)**
ENSBTAG00000038173	*HIST1H2BN*	chr23	Histone H2B type 1-K
ENSBTAG00000009548	*MAD2L1BP*	chr23	MAD2L1 binding protein
**ENSBTAG00000004150**	***NRG1***	**chr27**	**Neuregulin 1, ErbB signalling pathway**

Associated gene with inflammation and disease is shown in bold; Chr refers to chromosome.

^a^Gene name.

^b^WikiGene_description from Wikipedia website, https://en.wikipedia.org/wiki/Main_Page.
