# Supplementary material for: Combined analysis of DNA methylome and transcriptome reveal novel candidate genes with susceptibility to bovine Staphylococcus aureus subclinical mastitis
Source: Sci Rep. 2016 Jul 14;6:29390. doi: 10.1038/srep29390 (PMC4944166; doi:10.1038/srep29390)
Supplement: Supplementary Information [file srep29390-s1.pdf]

Combined analysis of DNA methylome and transcriptome reveal novel candidate genes with susceptibility to bovine *Staphylococcus aureus* subclinical mastitis

Minyan Song[1] Yanghua He[1,2] Huangkai Zhou[4] Yi Zhang[1] Xizhi Li[3] Ying Yu[1*]

[1] Key Laboratory of Animal Genetics, Breeding and Reproduction, Ministry of Agriculture & National Engineering Laboratory for Animal Breeding, College of Animal Science and Technology, China Agricultural University, 100193, Beijing, P.R. China

[2] Department of Animal & Avian Sciences, University of Maryland, College Park, Maryland, 20742, USA

[3] Beijing Sanyuan Breeding Technology Co. Ltd., Capital Agribusiness Group, Beijing, China

[4] Guangzhou Genedenovo Biotechnology Co., Ltd, Guangzhou, China

[*]Correspondence author:

Ying Yu, Ph.D, Associate Professor

Department of Animal Breeding and Genetics

China Agricultural University, Beijing, China.

Phone: 86-10-62734611

Fax: 86-10-62732439

Email：yuying@cau.edu.cn

Supplementary Figures:

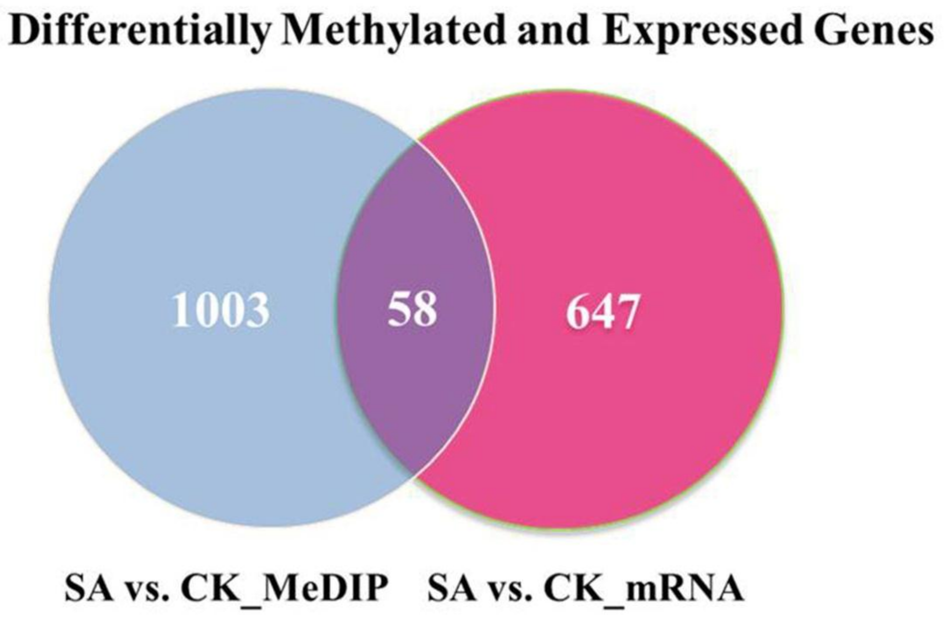

Figure S1 Differentially methylated and expressed genes that were unique or shared between two groups of SA and CK.

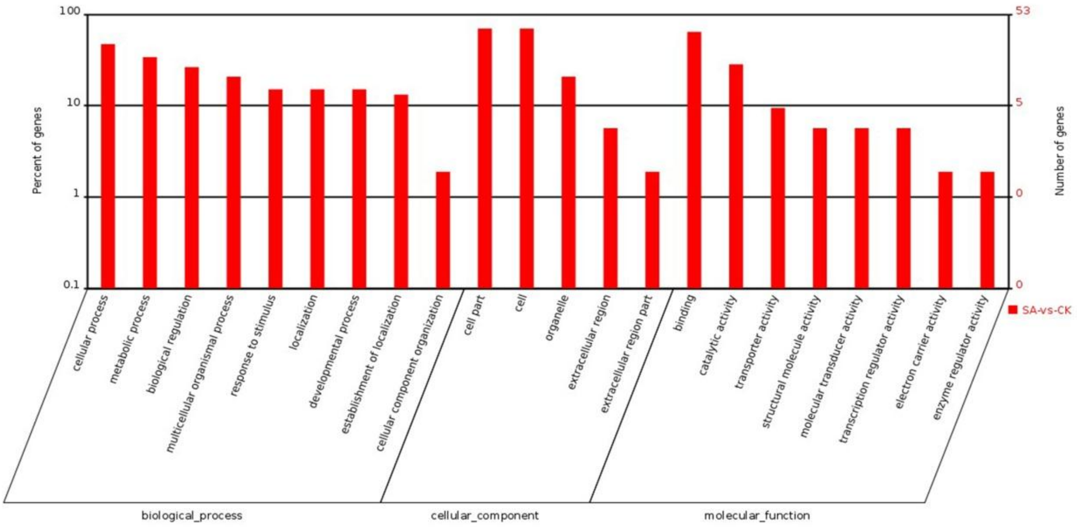

Figure S2 The functional enrichment of genes with differentially methylation and expression.

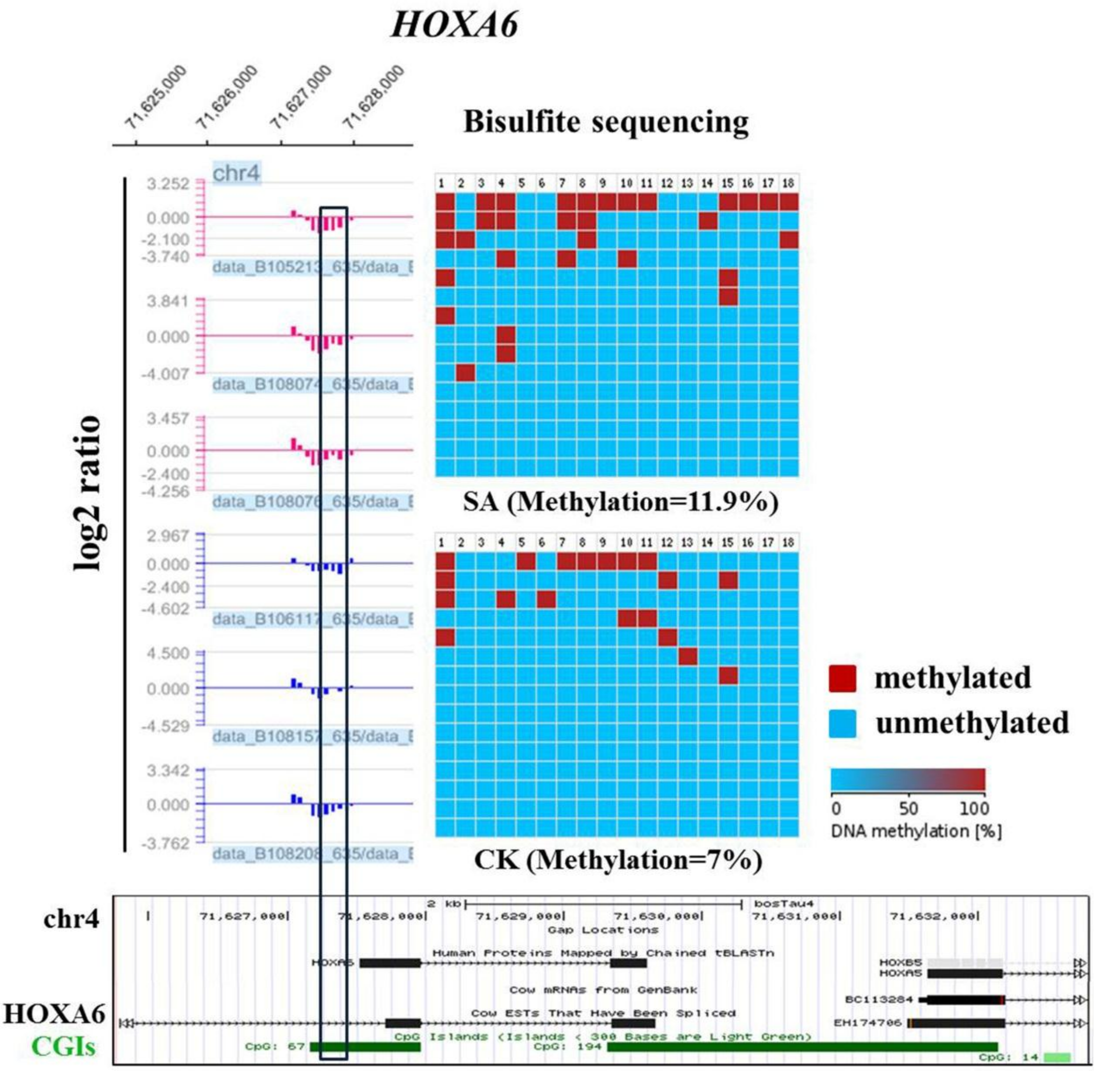

Figure S3. Bisulphite sequencing-PCR validation of *HOXA6* gene. Bisulphite cloning and sequencing results were shown with columns displayed CpG positions, while rows demonstrated the SA group (S1, S2 and S3) and CK group (C1, C2 and C3) individual clones.

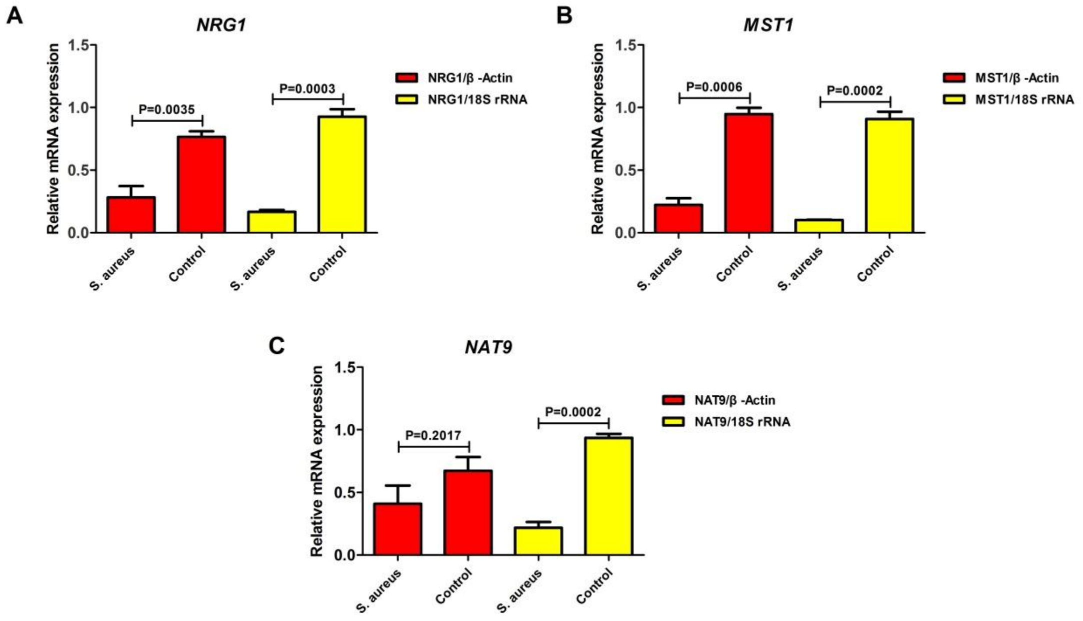

Figure S4 The mRNA expression levels of three differentially methylated and expressed genes in bovine mammary epithelial cell lines (Mac-T). A. *NRG1* gene. B. *MST1* gene. C. *NAT9* gene. Note: (1) $P<0.01$ and $P<0.05$ indicate highly significance difference and significance difference between the *S. aureu*s group and Control group, respectively. (2) *S. aureus* group indicates that Mac-T cell was stimulated by *S. aureus* at 6h. Control group means that the Mac-T cell was treated by DMEM at 6h. (3) The stability of three housekeeping genes were determined by NormFinder software package. The results showed that housekeeping genes *β-Actin* and *18S rRNA* are more stable than *GAPDH* for Mac-T cells. Therefore, *β-Actin* and *18S rRNA* were used to normalize the expression data of the target genes.

Supplementary Table Legends:

Table S1 Summary of DHI data in six samples.

(XLS)

Table S2 Peak distribution in different components of the cow genome.

(XLS)

Table S3 Summary of differentially methylated genes.

(XLS)

Table S4 Summary of differentially expressed genes.

(XLS)

Table S5 Summary of differentially methylated and expressed genes.

(XLS)

Table S6 Summary of primers used for PCR and qPCR.

(XLS)

Table S7 Summary of primers used for BSP.

(XLS)
